# Supplementary figures and images for: Vaccination with single plasmid DNA encoding IL-12 and antigens of severe fever with thrombocytopenia syndrome virus elicits complete protection in IFNAR knockout mice
Source: PLoS Negl Trop Dis. 2020 Mar 20;14(3):e0007813. doi: 10.1371/journal.pntd.0007813 (PMC7112229; doi:10.1371/journal.pntd.0007813)

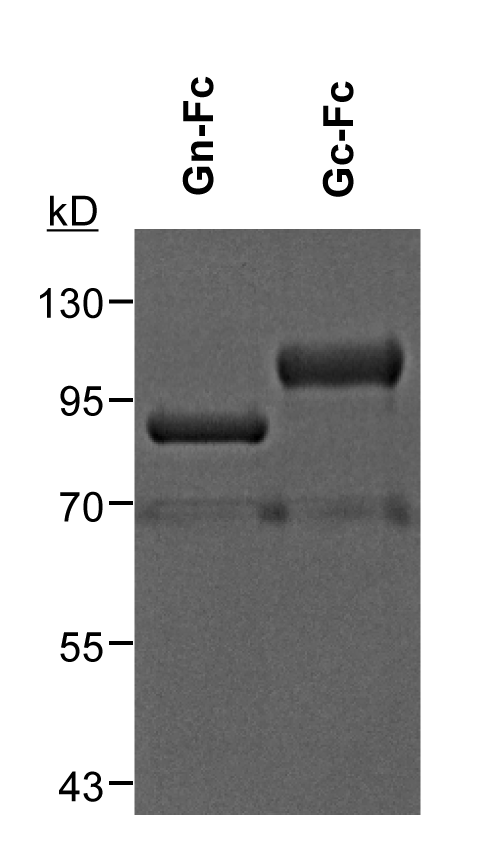

Supplement: S1 Fig — (TIF) [file pntd.0007813.s001.tif]

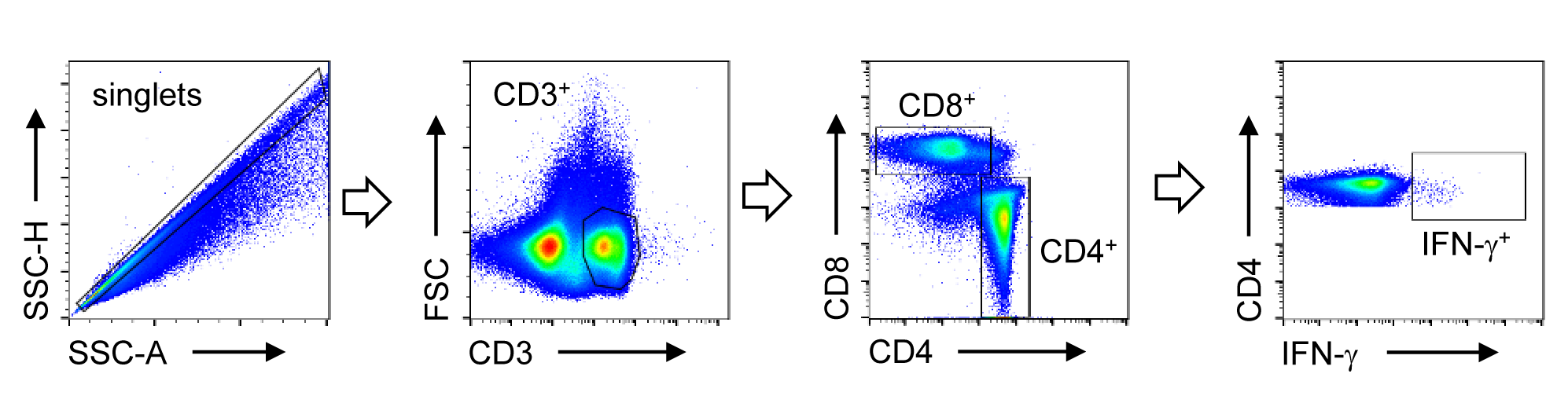

Supplement: S2 Fig — (TIF) [file pntd.0007813.s002.tif]

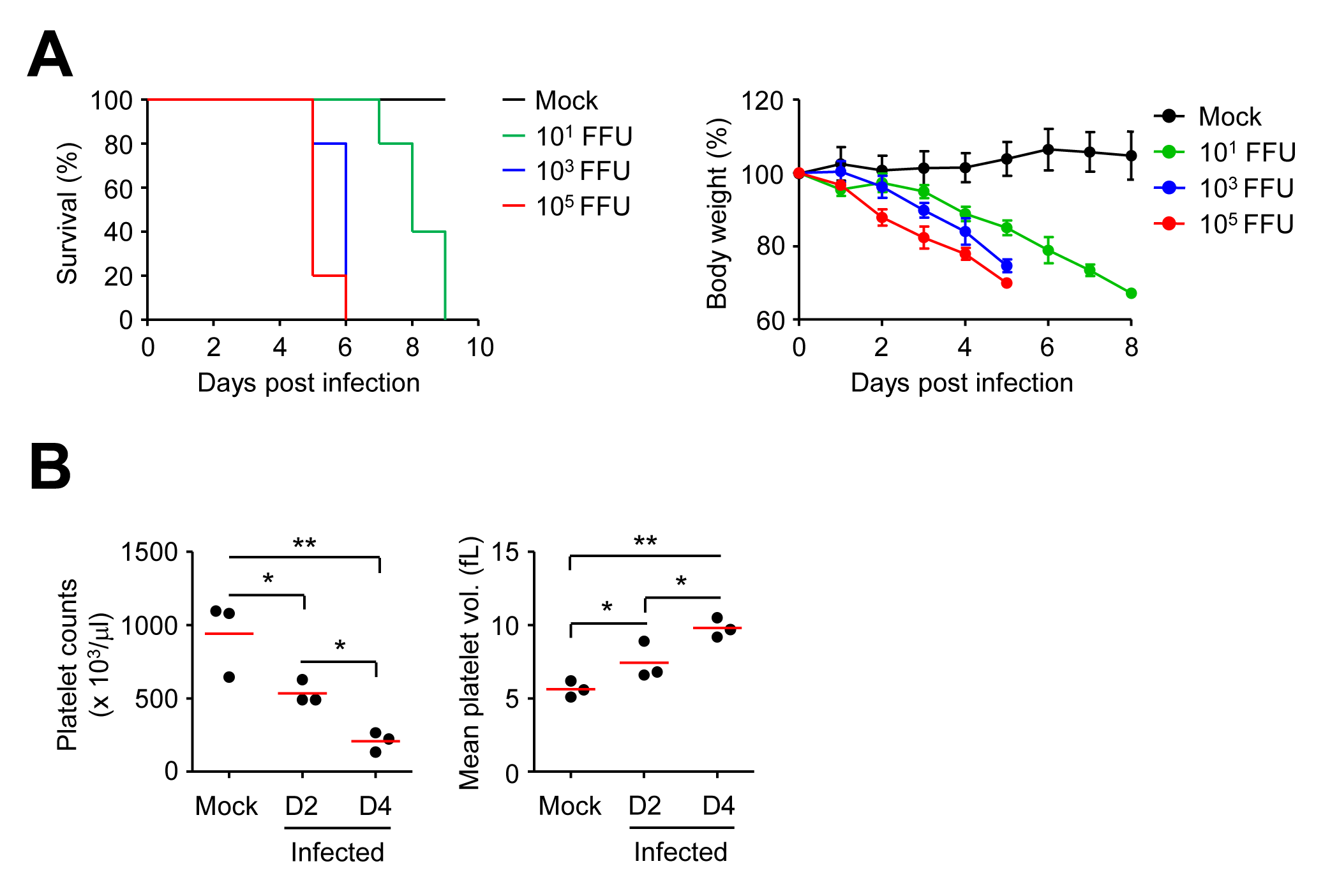

Supplement: S3 Fig — (A) IFNAR KO mice were subcutaneously infected with different doses (0, 101, 103, or 105 FFU/mouse) of SFTSV. The mice were monitored daily to assess survival rate (left) and body weight change (right) up to 10 d after infection when all the infected mice died. (B) The blood from the infected mice were collected and platelet counts and their volumes were examined by hematological analyzer. Red line: mean value, *: p < 0.05, **: p < 0.01, compared with mock-infected controls. (TIF) [file pntd.0007813.s003.tif]
